# Neuroprotective Effect of Salvianolic Acids against Cerebral Ischemia/Reperfusion Injury

**DOI:** 10.3390/ijms17071190

**Published:** 2016-07-22

**Authors:** Shuai Hou, Ming-Ming Zhao, Ping-Ping Shen, Xiu-Ping Liu, Yuan Sun, Jia-Chun Feng

**Affiliations:** 1 Department of Neurology and Neuroscience Center, the First Hospital of Jilin University, Changchun 130021, China; houshuai0310@163.com (S.H.); zhaommchn@163.com (M.-M.Z.); blush135@163.com (P.-P.S.); 2 Department of Neurology, the Center Hospital of Jilin City, Jilin 132000, China; liuxiuping12345@163.com; 3 Department of Neurology, Xuzhou Center Hospital, Xuzhou 221000, China; sysun12@126.com

**Keywords:** salvianolic acid, mitochondrial connexin43, phosphatidylinositol 3-kinase/protein kinase B, cerebral ischemia/reperfusion, astrocyte

## Abstract

This study investigated the neuroprotective effect of salvianolic acids (SA) against ischemia/reperfusion (I/R) injury, and explored whether the neuroprotection was dependent on mitochondrial connexin43 (mtCx43) via the phosphatidylinositol 3-kinase/protein kinase B (PI3K/AKT) pathway. In vitro, we measured astrocyte apoptosis, mitochondrial membrane potential, and also evaluated the morphology of astrocyte mitochondria with transmission electron microscopy. In vivo, we determined the cerebral infarction volume and measured superoxide dismutase (SOD) activity and malondialdehyde (MDA) content. Additionally, mtCx43, p-mtCx43, AKT, and p-AKT levels were determined. In vitro, we found that I/R injury induced apoptosis, decreased cell mitochondrial membrane potential (MMP), and damaged mitochondrial morphology in astrocytes. In vivo, we found that I/R injury resulted in a large cerebral infarction, decreased SOD activity, and increased MDA expression. Additionally, I/R injury reduced both the p-mtCx43/mtCx43 and p-AKT/AKT ratios. We reported that both in vivo and in vitro, SA ameliorated the detrimental outcomes of the I/R. Interestingly, co-administering an inhibitor of the PI3K/AKT pathway blunted the effects of SA. SA represents a potential treatment option for cerebral infarction by up-regulating mtCx43 through the PI3K/AKT pathway.

## 1. Introduction

Cerebral infarction remains a leading cause of morbidity and mortality in the developed world [1]. Intravenous thrombolysis is currently the most effective treatment for patients with acute cerebral infarction. However, due to the moderate recanalization rate, the limited time window, and contraindications for thrombolysis, only a minority of patients can receive the treatment [2]. Other treatment avenues involve antiplatelet aggregation, neurotrophic medicine, and improvement of blood circulation in ischemic penumbra. Therefore, the development of novel therapeutic options is urgently needed to limit injury after cerebral infarction.

Salvianolic acids (SA) are water-soluble compounds extracted from *Radix Salvia miltiorrhiza* (Danshen), a perennial herb used in traditional Chinese medicine to improve blood flow. The two most common types of SA are salvianolic acid A (SalA) and B (SalB). Previous studies have shown that SalA confers protection against concanavalin A-induced liver injury [3], and that SalB is an effective treatment component for hepatic fibrosis [4]. Additionally, salvianolate has been widely recognized for its cardiovascular benefits [5]. However, the use of SA to treat cerebrovascular diseases has only recently begun to be investigated.

A number of recent studies have focused on a potential role of hemichannels in pathological damage, which constitute one half of a gap junction [6,7]. Hemichannels are not only found in the sarcolemma of cells, but also in the inner mitochondrial membrane where they regulate ionic homeostasis and respiration [8]. Each hemichannel consists of six connexins, which are a family of gap junction proteins. Connexin43 (Cx43) is the most prominently expressed in astrocytes in the gap junction proteins and has been shown to be critical for astrocytes function [9]. In particular, previous studies have reported that Cx43 localized in the mitochondria has a number of protective effects in cardiocytes [10,11]. Additionally, astrocytes exert a protective role in cerebral ischemia/reperfusion (I/R) injury to preserve the function of neuron [12]. Therefore, we hypothesized that mitochondrial connexin43 (mtCx43) in astrocytes could play an important role in neuroprotective effect of SA against cerebral infarction.

Phosphatidylinositol 3-kinase/protein kinase B (PI3K/AKT) pathway plays a crucial role in proliferation, differentiation, inflammation, and apoptosis [13]. Reduction of apoptosis could be critical in ischemic penumbra, in which cells are potentially salvageable after stroke [14]. Additionally, some studies suggest that the protection against I/R injury in cardiocytes is related to the up-regulation of Cx43 by the activation of PI3K/AKT pathway [15].

In the present study, we examined the effect of SA on brain tissue in vivo and astrocytes in vitro following cerebral I/R injury. Furthermore, we investigated whether SA elicited beneficial effects by activating mtCx43 through the PI3K/AKT pathways.

## 2. Results

### 2.1. Effect of SA on Astrocytic Apoptosis Following Oxygen-Glucose Deprivation (OGD) Injury

**Figure 1:**
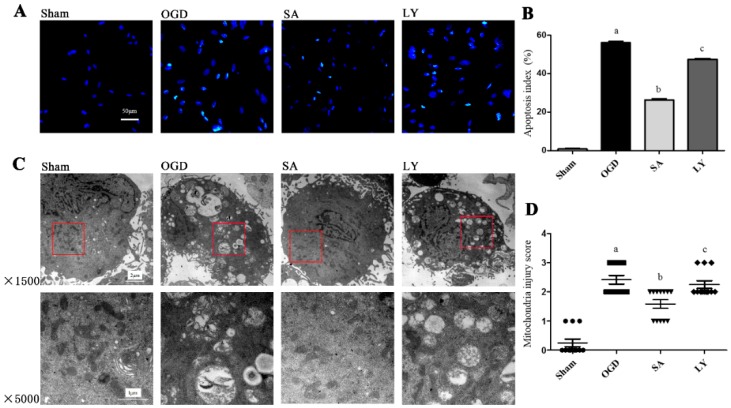
The effects of SA and LY on cell apoptosis and structure following OGD: (**A**) representative images of cell apoptosis by TUNEL staining; (**B**) analysis of the apoptotic index; (**C**) representative images of mitochondrial ultrastructure by TEM, where areas surrounded by red boxes in the above images correspond to the magnified images below; and (**D**) analysis of mitochondrial injury using Flameng scores. *n* = 3 in each group. ^a^ *p* ≤ 0.01 vs. Sham; ^b^ *p* ≤ 0.05 vs. OGD; ^c^ *p* ≤ 0.05 vs. SA. SA: salvianolic acids; LY: LY 294002; OGD: oxygen glucose deprivation; TEM: transmission electron microscopy; TUNEL: terminal deoxynucleotidyl transferase dUTP nick end labeling.

We detected astrocytic apoptosis by terminal deoxynucleotidyl transferase dUTP nick end labeling assay (TUNEL) staining, as shown in Figure 1A,B. All positive nuclei were karyopyknotic and stained with green fluorescence. Astrocytes in the OGD group exhibited a significant increase in positive nuclei compared to that in the sham group (*p* ≤ 0.01). SA treatment dramatically attenuated apoptosis compared to sham group; however, this reduction was significantly blunted by the PI3K/AKT pathway inhibitor LY 294002 (LY) treatment (*p* ≤ 0.05).

### 2.2. Effect of SA on Ultrastructural Damage of Astrocyte Mitochondria

As shown in Figure 1C,D, we examined the ultrastructure of mitochondria by transmission electron microscopy (TEM). In the sham group, the mitochondria were circular or oval-shaped and the double membrane was clear, with obvious cristae. In the OGD group, the mitochondria were obviously injured as the double membranes were dimmed, the cristae were vague, and vacuolar degeneration was marked; some mitochondria were swollen and destroyed. Compared to those in the OGD group, there were fewer signs of damage in the mitochondria of the SA group (*p* ≤ 0.05). Conversely, damage to mitochondria in the LY group was more apparent than those in the SA group (*p* ≤ 0.05).

### 2.3. Effect of SA on Astrocyte Mitochondrial Membrane Potential (MMP)

**Figure 2:**
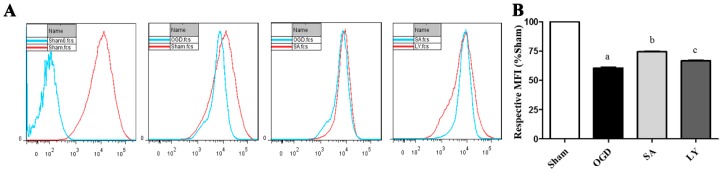
The effects of SA and LY on astrocyte MMP following OGD: (**A**) representative flow cytometry images of MMP observed with the Rh 123 probe; and (**B**) MMP analysis is calculated as a percent relative to the mean fluorescence intensity of the sham group. *n* = 3 in each group. ^a^ *p* ≤ 0.05 vs. Sham; ^b^ *p* ≤ 0.05 vs. OGD; ^c^ *p* ≤ 0.05 vs. SA. SA: salvianolic acids; LY: LY 294002; OGD: oxygen glucose deprivation; MMP: mitochondrial membrane potential; Rh 123: Rhodamine 123.

In order to investigate a possible cytoprotective effect of SA on OGD-induced mitochondria damage in astrocytes, we determined cell MMP by Rhodamine 123 (Rh 123) staining. As shown in Figure 2A, MMP was obviously decreased by OGD injury compared to the sham group (*p* ≤ 0.05). However, MMP in the SA group was higher compared to that in the OGD group (*p* ≤ 0.05). Interestingly, LY attenuated the neuroprotection induced by SA.

### 2.4. Infarct Volume and Neurological Deficit Scores after Middle Cerebral Artery Occlusion (MCAO)

**Figure 3:**
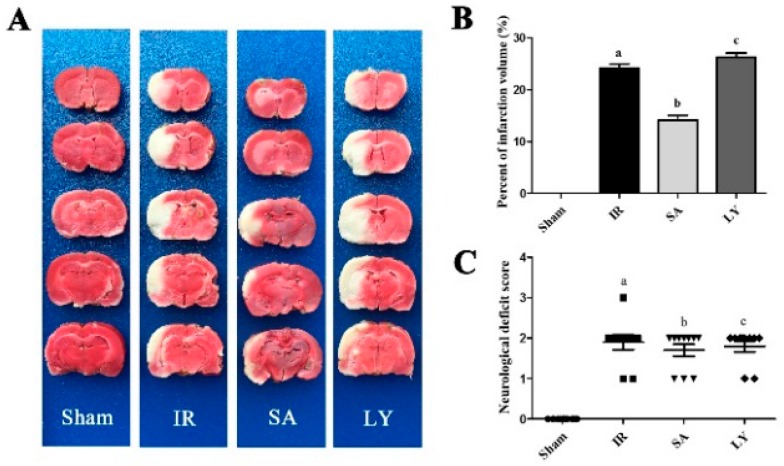
The effects of SA and LY on infarction volume and neurological deficits in rats following MCAO: (**A**) representative images of cerebral infarction after 2 h of MCAO and a 12 h reperfusion in rat brains by 2,3,5-triphenyltetrazolium chloride (TTC) staining; (**B**) analysis of infarct volume, which is calculated as a percent relative to total cerebral volume (*n* = 3 in each group. ^a^ *p* ≤ 0.01 vs. Sham; ^b^ *p* ≤ 0.05 vs. IR; ^c^ *p* ≤ 0.05 vs. SA); and (**C**) neurological deficits analysis (*n* = 10 in each group. ^a^ *p* ≤ 0.01 vs. Sham; ^b^ *p* > 0.05 vs. IR; ^c^ *p* > 0.05 vs. SA). SA: salvianolic acids; MCAO: middle cerebral artery occlusion; LY: LY 294002; TTC: 2,3,5-triphenyltetrazolium chloride; IR: ischemia-reperfusion. Additionally, we investigated neurological deficits following MCAO, as shown in Figure 3C. All rats in the sham group were scored zero. However, there was a significant deterioration in neurological functioning in the I/R group, compared to the sham group (*p* ≤ 0.01). No improvement was noted in the scores in the SA groups compared to the I/R group after surgery.

In order to evaluate the effect of SA in vivo, we examined infarct volume after MCAO. The respective volumes of rat cerebral infarctions are shown in Figure 3A. Injection of SA before MCAO significantly reduced the infarct volume, compared to the I/R group (*p* ≤ 0.05). However, LY significantly attenuated this effect (*p* ≤ 0.05).

### 2.5. Effect of SA on Superoxide Dismutase (SOD) Activity and Malondialdehyde (MDA) Content

The generation of reactive oxygen species during cerebral I/R is widely regarded as the initial step after stroke. Therefore, we investigated SOD activity and MDA content in the rat cerebral cortex following I/R injury, as shown in Table 1. Compared to the sham group, SOD activity was significantly decreased and MDA levels were elevated in the I/R group (*p* ≤ 0.05). Interestingly, pre-treatment with SA increased SOD activity and decreased MDA levels after ischemia (*p* ≤ 0.05). Conversely, LY blocked the beneficial effects of SA.

### 2.6. Effect of SA on mtCx43, p-mtCx43, AKT, and p-AKT Expression

**Figure 4:**
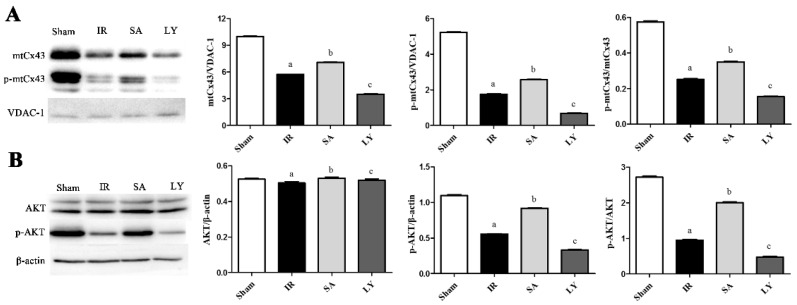
The effects of SA on the expression of mtCx43, p-mtCx43, AKT, and p-AKT in the rat cerebral cortex after I/R injury: (**A**) Western blotting showed mtCx43, p-Cx43 and p-mtCx43/p-Cx43 were up-regulated in SA group after MCAO. These effects were reduced by LY; (**B**) Western blotting showed p-AKT and p-AKT/AKT were up-regulated in SA group after MCAO. These effects were reduced by LY. *n* = 3 in each group, ^a^ *p* ≤ 0.01 vs. Sham; ^b^ *p* ≤ 0.05 vs. IR; ^c^ *p* ≤ 0.05 vs. SA. There was no difference in AKT level. I/R: ischemia-reperfusion; SA: salvianolic acids; mtCx43: mitochondrial connexin 43; Cx43: connexin 43; p-: phosphorylated; MCAO: middle cerebral artery occlusion; AKT: protein kinase B; VDAC-1: voltage-dependent anion channel; LY: LY 294002.

We measured Cx43 levels in mitochondria and AKT levels in the cortex, as shown in Figure 4. In mitochondrial proteins, voltage-dependent anion channel (VDAC-1) was the loading control. Cerebral I/R injury markedly decreased the amount of both mtCx43 and p-mtCx43, and reduced the p-mtCx43/mtCx43 ratio, compared to the sham group (*p* ≤ 0.05 for all). In the SA group, these levels were comparatively increased (*p* ≤ 0.05), and these effects could be reversed by treatment with LY (*p* ≤ 0.05). The results for p-AKT and p-AKT/AKT ratio in the cortex were similar to those for mtCx43, while AKT level was not changed significantly among the different groups.

## 3. Discussion

In the current study, we investigated the neuroprotective effects of SA in a stroke model both in vivo and in vitro, and provided a novel mechanism by which SA ameliorates cerebral I/R injury. We found that SA improved damages following I/R injury, including reduction of cortex infarct volume, prevention of apoptosis, and increase of MMP. Additionally, damage to the mitochondria structure and function were all attenuated by SA. Interestingly, I/R injury lead to a decreased expression of mtCx43, which was also attenuated by SA, indicating that mtCx43 may play a role in the neuroprotection induced by SA. Importantly, the neuroprotective effects of SA, as well as the increase in mtCx43, could be blocked by inhibiting the PI3K/AKT pathway with LY. Taken together, our results suggest that SA represents a potential treatment option for cerebral ischemic infarctions, and works through a mechanism that is dependent on mtCx43 through the PI3K/AKT pathway.

Previous studies have shown that SA can reduce intracellular oxidative stress [16,17], improve circulation in the smaller arteries [18], and protect cardiomyocytes from peroxidation [19]. In addition, SA attenuates endothelial-leukocyte adhesion molecule expression on vascular endothelial cells [20]. Similarly, Danshen has been shown to improve cardiac function in hypertrophy, in which case the effects were partially correlated with Cx43 expression [21]. However, few studies have focused on the effect of SA on mtCx43 following cerebral I/R injury. The most devastating consequences of I/R injury result in glutamate excitotoxicity, calcium overload, and generation of reactive oxygen species [22], and mitochondria are critical to many of these processes. Therefore, we investigated for signs of damage in both the ischemic penumbra, and the mitochondria structure and function. We found that SA exerted a protective effect in both the mitochondria and ischemic penumbra during I/R injury.

Additionally, mtCx43 was down-regulated following I/R injury. Activated mtCx43 has been shown to inhibit the permeability transition pore, leading to mitochondrial demise and cell death [8]. We had observed that astrocytes subjected to OGD injury had a decreased expression of Cx43. Importantly, these findings corresponded with our in vivo experiments, in which mtCx43 expression was significantly diminished after I/R injury. This decrease was rescued by treatment with SA. Taken together, these findings indicate that SA ameliorates cerebral I/R injury by increasing mtCx43 expression.

In the central nervous system, the PI3K/AKT pathway is an important neuroprotective and anti-apoptotic pathway. Suppression of AKT activity has been associated with the neuronal death that follows I/R injury [23]. Moreover, a previous study showed that AKT is activated by Ser473 phosphorylation [24]. Our results showed that I/R injury decreased the expression of p-AKT, but this effect did not occur at the AKT level, which is consistent with previous work [15]. Similarly, Cx43 is activated by phosphorylationvia AKT activity [25]. In this study, we observed that the untreated MCAO rats had a significant decrease in p-mtCx43 expression. Furthermore, we found that inhibition of PI3K/AKT pathway attenuated the p-mtCx43 increase by treatment of SA. Thus, SA may regulate mtCx43 through PI3K/AKT signaling, implicating this pathway in the protective effects of SA on cerebral I/R injury.

In conclusion, these findings provide novel insight into a potential method of protection against cerebral infarction, as well as the mechanism by which SA exerts its neuroprotective effects. Future work may focus on verifying effects reported herein, particularly through the use of Cx43 knockout mice. Moreover, other pathways are likely involved in this process and future studies might address this possibility.

## 4. Materials and Methods

### 4.1. Animals and Drugs

All experiments were performed with either newborn or adult male Wistar rats, weighing 250–280 g. All animal experiments were performed with the approval of the Institutional Animal Care and approved by the Animals Ethics Committee of Jilin University of China (10 February 2014, NO. 2014-277).

SA used in this study is a commercially available drug, which is provided by Tianjin Tably Pride Pharmaceutical Company (Tianjing, China) (Z20110011). It is a mixture and consisted of SalB, SalE, SalD and some other acids. The mixture has the advantages of low resistance and low toxicity.

### 4.2. Isolation and Culture of Rat Astrocytes

Astrocytes were obtained from newborn rats as described previously [26]. Microglia and oligodendrocytes were removed from confluent primary glial cultures by shaking. The purity of astrocytes exceeds 95% as verified by immunohistochemistry staining for the astrocyte specific maker, glial fibrillary acidic protein (GFAP) (Abcam, Cambridge, MA, USA, ab7260).

In vitro, OGD injury is a representative model of I/R injury. Astrocyte cultures were divided into four experimental groups as follows: (1) the Sham group, stimulated with phosphate buffered solution (PBS) and not subjected to injury; (2) the OGD group, stimulated with PBS during OGD injury; (3) the SA (Tianjin Tably Pride Pharmaceutical Co., Ltd., Tianjing, China) group, stimulated with SA (8 μg/mL) during OGD injury; and (4) the LY group, stimulated with LY (20 μM) (Abcam, Cambridge, MA, USA) and SA during OGD injury.

### 4.3. OGD Injury to Astrocytes

Astrocytes were washed with PBS three times and re-suspended in DMEM/F12 medium without glucose. Cells were allowed to grow in a hypoxia chamber with a mixture of 0.1% O_2_, 94.9% N_2_ and 5% CO_2_ for 12 h. After hypoxia, the cells were transferred back to normal medium and incubated for 6 h in an atmosphere of 95% air and 5% CO_2_.

### 4.4. Analysis of Astrocyte Mitochondria Ultrastructure by Transmission Electron Microscopy

Astrocytes in each group were treated with 2.5% glutaraldehyde for 24 h after harvest. Slices were collected to obtain ultrathin sections. Sections were mounted on single-hole copper grids and multi-hole grids, stained with uranyl acetate and citric acid lead, and examined under a TEM. Injury to the mitochondria was evaluated by Flameng scores [27].

### 4.5. TUNEL Staining to Measure Astrocyte Apoptosis

Astrocytes apoptosis was analyzed by terminal deoxynucleotidyl transferase dUTP nick end labeling assay (TUNEL, Roche Molecular Biochemicals, Mannheim, Germany) according to the manufacturer’s instructions. The nucleus was stained by 4,6-diamidino-2-phenylindole (DAPI) and observed with a laser scanning confocal microscope (LSCM). The apoptotic index was determined as the number of positive cells/the total number of cells × 100%.

### 4.6. Detection of Mitochondrial Membrane Potential

MMP was quantitatively analyzed by the Rh 123 (Sigma, St. Louis, MO, USA), which is a cell permeable, cationic fluorescence probe. Astrocytes were washed with PBS three times, and then incubated with 1 μM Rh 123 in the dark for 30 min at 37 °C. Then, cells were washed in PBS. Mean fluorescence intensity (MFI) of Rh 123-labeled cells was analyzed by flow cytometry using 488 nm excitation, and at least 1 × 105 events per sample were acquired.

### 4.7. Middle Cerebral Artery Occlusion Model

Cerebral infarction was induced using the MCAO model similar to previously described [28]. Following artery occlusion rats were kept in a cage for 2 h, and then the suture was removed. After MCAO, rats were allowed free access to food and water for 12 h. Sixty rats were randomly divided into four groups (*n* = 15 in each group) as follows: (1) the sham group, given an injection of 0.9% normal saline and was not subjected to MCAO; (2) the I/R group, given an injection of 0.9% normal saline; (3) the SA group, given an injection of SA (10 mg/kg); and (4) the LY group, given an injection of LY (0.3 mg/kg), and after 10 min, SA was injected as previous. All injections were given intraperitoneally 30 min prior to MCAO.

### 4.8. Neurological Evaluation

Neurological evaluations were performed by researchers blinded to experimental group, following reperfusion. Evaluations were performed using a modified form [28] as follows: (0) no deficits; (1) difficulty fully extending the contralateral forelimb; (2) unable to extend the contralateral forelimb; (3) mild circling to the contralateral side; (4) severe circling; and (5) falling to the contralateral side.

Following evaluations, rats were anesthetized with 10% chloral hydrate and decapitated. The ischemic cerebral cortex was rapidly removed and stored at −70 °C until use for Western blotting and analysis of SOD activity and MDA content.

### 4.9. TTC Staining

2,3,5-triphenyltetrazolium chloride (TTC, Sigma, St. Louis, MO, USA) staining was used to visualize the ischemic infarction. All brains were sliced into 2 mm sections and each slice was incubated for 20 min in a 2% solution of TTC at room temperature, and then fixed in 4% paraformaldehyde. Infarct size was determined with image analysis software.

### 4.10. Mitochondria Isolation

Cerebral cortical mitochondria were isolated by differential centrifugation using a functional mitochondria isolation kit (Nanjing Jiancheng Bioengineering Institute, Nanjing, China), which was mentioned in our previous study [29]. The isolated mitochondria were stored at −70 °C.

### 4.11. Detection of SOD Activity and MDA Content

Tissue samples were homogenized in ice cold saline, and both SOD activity and MDA content were measured using commercially available detection kits (Nanjing Jiancheng Bioengineering Institute, Nanjing, China), according to the manufacturers’ instructions. Briefly, SOD activity was assessed using the xanthine oxidase method, and MDA content was determined with the thiobarbituric acid method. The samples were analyzed with a spectrophotometer.

### 4.12. Western Blot Analysis

Cerebral ischemic cortex or mitochondria were homogenized in lysis buffer. Each sample (50 μg) was loaded onto a 12% sodium dodecyl sulfate-polyacrylamide gel electrophoresis apparatus. Then, the membranes were blocked with antibodies to Cx43 (Abcam, Cambridge, MA, USA, ab11369, 1:2000), p-Cx43 (Abcam, Cambridge, MA, USA, ab30559, 1:2000), AKT (Abcam, Cambridge, MA, USA, ab25893, 1:500), p-AKT (Abcam, Cambridge, MA, USA, ab81283, 1:500), voltage-dependent anion channel (Abcam, Cambridge, MA, USA, ab34726, VDAC-1; 1:2000), and β-actin (Abcam, Cambridge, MA, USA, ab8227, 1:1000) overnight at 4 °C. The membranes were incubated with horseradish peroxidase-conjugated secondary antibody (Bioss, Beijing, China) in blocking solution for 1 h. Immunoblots were scanned, and protein bands were quantified with Quantitation One software. Relative abundance was obtained by normalizing the density of proteins against that of β-actin or VDAC-1.

### 4.13. Statistical Analysis

All data are expressed as mean ± standard deviation of multiple experiments. ANOVA with Fisher’s Least Significant Difference (LSD) post tests were used to compare multiple groups by SPSS 16.0 software (SPSS Company, Chicago, USA). Statistical significance was detected at the 0.05 level.

## Figures and Tables

**Table 1:** SOD activity and MDA content in rats following MCAO.

Group (*n* = 6 in Each)	SOD Activity (U/mg Protein)	MDA Content (mmol/mg Protein)
Sham	118.9 ± 2.3	6.1 ± 0.3
IR	91.0 ± 2.5 ^a^	10.8 ± 0.6 ^a^
SA	137.6 ± 1.9 ^b^	8.3 ± 0.3 ^b^
LY	96.3 ± 2.4 ^c^	9.6 ± 0.4 ^c^

^a^ *p* ≤ 0.05 vs. Sham; ^b^ *p* ≤ 0.05 vs. I/R; ^c^ *p* ≤ 0.05 vs. SA. *n* = 3 in each group. Data are presented as mean ± standard deviation. IR: ischemia-reperfusion; SA: salvianolic acids; LY: LY 294002; SOD: superoxide dismutase; MDA: malondialdehyde; MCAO: middle cerebral artery occlusion.

## Abbreviations

I/R	ischemia-reperfusion
Cx43	connexin 43
mtCx43	mitochondrial connexin 43
MCAO	middle cerebral artery occlusion
PI3K/AKT	phosphatidylinositol 3-kinase/protein kinase B
SA	salvianolic acids
SalB	salvianolic acid B
SalA	salvianolic acid A
LY	LY 294002
OGD	oxygen glucose deprivation
TTC	2,3,5-triphenyltetrazolium chloride
DAPI	4,6-diamidino-2-phenylindole
TUNEL	terminal deoxynucleotidyl transferase dUTP nick end labeling
MMP	mitochondrial membrane potential
Rh 123	Rhodamine 123
p-	phosphorylated
VDAC-1	voltage-dependent anion channel
SOD	superoxide dismutase
MDA	malondialdehyde
TEM	transmission electron microscopy
